# The Role of Inflammation, Hypoxia, and Opioid Receptor Expression in Pain Modulation in Patients Suffering from Obstructive Sleep Apnea

**DOI:** 10.3390/ijms23169080

**Published:** 2022-08-13

**Authors:** Piotr Kaczmarski, Filip Franciszek Karuga, Bartosz Szmyd, Marcin Sochal, Piotr Białasiewicz, Dominik Strzelecki, Agata Gabryelska

**Affiliations:** 1 Department of Sleep Medicine and Metabolic Disorders, Medical University of Lodz, 92-215 Lodz, Poland; 2 Department of Neurosurgery, Spine and Peripheral Nerves Surgery, Medical University of Lodz, 90-549 Lodz, Poland; 3 Department of Affective and Psychotic Disorders, Medical University of Lodz, 90-419 Lodz, Poland

**Keywords:** pain, OSA, obstructive sleep apnea, inflammation, hypoxia, opioids, nociceptors

## Abstract

Obstructive sleep apnea (OSA) is a relatively common disease in the general population. Besides its interaction with many comorbidities, it can also interact with potentially painful conditions and modulate its course. The association between OSA and pain modulation has recently been a topic of concern for many scientists. The mechanism underlying OSA-related pain connection has been linked with different pathophysiological changes in OSA and various pain mechanisms. Furthermore, it may cause both chronic and acute pain aggravation as well as potentially influencing the antinociceptive mechanism. Characteristic changes in OSA such as nocturnal hypoxemia, sleep fragmentation, and systemic inflammation are considered to have a curtailing impact on pain perception. Hypoxemia in OSA has been proven to have a significant impact on increased expression of proinflammatory cytokines influencing the hyperalgesic priming of nociceptors. Moreover, hypoxia markers by themselves are hypothesized to modulate intracellular signal transduction in neurons and have an impact on nociceptive sensitization. Pain management in patients with OSA may create problems arousing from alterations in neuropeptide systems and overexpression of opioid receptors in hypoxia conditions, leading to intensification of side effects, e.g., respiratory depression and increased opioid sensitivity for analgesic effects. In this paper, we summarize the current knowledge regarding pain and pain treatment in OSA with a focus on molecular mechanisms leading to nociceptive modulation.

## 1. Introduction

In recent decades, obstructive sleep apnea (OSA) has become a serious societal problem, as it is one of the most prevalent sleep disorders in the world [1]. OSA is a chronic condition caused by the collapse of upper airways during sleep, resulting in nocturnal hypoxemia and sleep fragmentation. The increasing prevalence of OSA is worrying in itself, although the numerous conditions accompanying the disorder have to be taken into consideration while providing the best holistic care for OSA patients. The most common comorbidities can be divided into cardiovascular disorders (e.g., hypertension and ischemic heart disease) and metabolic disorders (e.g., type II diabetes, insulin resistance, or metabolic syndrome) [2,3]. Interaction between pain and process occurring during OSA is a relatively new topic of researchers’ interest. However, currently, there is more and more evidence, both at a clinical as well as a molecular level, that sleep-disordered breathing may be responsible for the aggravation and/or modulation of both chronic and acute pain. The link between pain and sleep-disordered breathing is a widely discussed problem in terms of analgesic therapy for OSA patients [4] and the influence of chronic opioid therapy on the onset of OSA [5], as well as the possible aggravation of chronic and acute pain in patients suffering from OSA. The exact mechanism of this phenomenon has not been yet fully understood, though it is possibly related to two main pathophysiological mechanisms of OSA—nocturnal hypoxemia and sleep fragmentation [6,7,8].

### 1.1. Pathophysiology of OSA

OSA is a sleep breathing disorder caused by airflow cessation/reduction in upper airways, which manifests as loud snoring, arousals (brief awakenings from sleep), nocturnal intermittent blood gas disturbances, and changes in sympathetic activation. Disruption of nocturnal ventilation leads to a cyclical breathing pattern and sleep fragmentation. Patients with OSA balance between wakefulness and sleep, leading to daytime sleepiness, decreased quality of sleep, and overall lower quality of life [9].

The upper airways’ ability to collapse may result from anatomical variations in the upper airways as well as from dilatator muscle activity and reflex responsiveness. The interaction between compromised pharyngeal anatomy and decreased activity of upper-airway dilatators is a key factor involved in OSA pathophysiology. It is described that dilatator muscle tone is reduced during sleep onset, which may evoke the collapse of upper airways and respiratory events in OSA patients [10,11]. Additionally, the activity of the main upper-airway dilatator—the genioglossus muscle—is increased by a local mechanoreceptor reflex responsive to negative pharyngeal pressure. There is some evidence that this reflex may be impaired during sleep, leading to decreased genioglossus muscle activity and therefore increased collapsibility of upper airways [12]. The collapse of upper airways and hypopnea/apnea events result in changes in the blood–gas balance—nocturnal hypoxemia and hypercapnia.

The episodes of hypopnea/apnea during sleep lead to arousal—the most important protective mechanism for upper airway reopening [13]. Arousal is associated with increased upper airway muscle activity, resulting in restored ventilation. Due to impaired ventilation regulatory mechanisms, OSA patients have lower possibility of maintaining open airways during sleep without arousal. Arousal threshold (ArTH) in OSA is defined as the level of ventilatory drive preceding electroencephalogram arousal. Low ArTH is a common phenomenon in OSA and may be responsible for sleep fragmentation [14]. Lowered ArTH prevents the increase in respiratory drive and laryngeal dilatator muscle activation. These changes lead to sleep fragmentation and aggravation of sleep apnea episodes. Respiratory events in OSA tend to occur during decreased respiratory drive.

Ventilation regulatory disturbances are essential in OSA pathogenesis, as ventilatory control plays an essential role in maintaining stable ventilation during sleep in healthy humans. In OSA patients, ventilatory control system sensitivity is increased, which means that respiratory stimulus evokes increased ventilatory response. Increased ventilatory response in OSA may lead to various changes in nocturnal breathing pattern, e.g., increased response to hypercapnia may result in a decrease in PaCO_2_ below the apnea threshold and result in apnea during next the sleep period [9].

The described mechanisms in OSA result in sleep fragmentation and nocturnal hypoxemia. These two changes in OSA patients may be involved in the development of a number of OSA comorbidities (e.g., glucose metabolism disturbances) and molecular changes (e.g., inflammatory processes, pain sensitization) [15,16,17].

### 1.2. General Pain Mechanisms

Pain is defined as the process by which an unpleasant, harmful stimulus from the periphery is carried through the spinal cord and to various areas of the central nervous system (CNS), causing a physiological sensation of pain and the associated negative emotional reaction, ultimately resulting in the sensation of pain [18]. The main pain classification is based on the duration and etiology of pain. Acute pain is described by the International Association for the Study of Pain as “pain of recent onset and probable limited duration. It usually has an identifiable temporal and causal relationship to injury or disease” [19,20]. The timeframe of acute pain duration has been suggested as 12 weeks or no longer than the time of healing of initial insult [21]. Chronic pain is commonly defined as pain lasting beyond the time of primary injury. It is mostly mediated by C fibers and may include elements of central sensitization [22]. Pain may also be divided in accordance with the location of stimulus initiation. Nociceptive pain is evoked due to the activation of nociceptors as a response to actual or potential damage to nonneural tissue [22,23]. The injury of the peripheral nerve or central neurons may result in neuropathic pain. The pathogenesis of neuropathic pain includes peripheral and central sensitization mechanisms, alteration in ion channels, and pain modulation resulting in chronic pain conditions [23,24]. The pathway of painful stimulus to the sensation of pain consists of transduction, transmission, modulation, and perception. Stimulation of nociceptors in peripheral axons of primary afferent neurons leads to the transduction of noxious stimuli into an electrical impulse. Transduction depends on the activation of neuronal receptors and membrane depolarization in response to chemical, mechanical and thermal stimuli [22]. If the generation’s potential magnitude is higher than the pain threshold, the action potential is transmitted through an afferent neuron [25].

### 1.3. Pain Transduction

Primary afferent neurons contain several surface proteins that are able to transduce noxious stimuli. There are three classes of transducer surface proteins, which include: ion channels, metabotropic G protein-coupled receptors (GPCRs), and receptors for neurotrophins or cytokines [25]. Transduction of stimuli might take place through direct action on the transducer or indirectly through mediators released by the injured area.

Among ion channels, the most common transducers are the transient receptor potential (TRP) channel families [26,27]. The family of TRP channels consists of six subfamilies. The best-known subfamily of TRP is the thermosensitive group of ion channels subfamily V (TRPV) [26]. A member of this subfamily is the transient receptor potential cation channel subfamily V member 1 (TRPV1), which apart from thermal nociception, is activated by capsaicin and may be potentiated by inflammatory mediators (e.g., bradykinin and ATP), and is considered to be an integrator of inflammatory pain pathways [26,27]. Another channel from TRP family is the transient receptor potential ankyrin 1 (TRPA1). Both TRPA1 and TRPV1 play an important role in pain transduction and in neurogenic inflammation through sensory neuron activation [28]. Almost all TRPA1-expressing neurons also express TRPV1; additionally, TRPA1 channel functions depend on coexistence with TRPV1, and only coexisting channels have activating function on sensory neurons [28]. TRPA1 channels are activated by low temperatures, chemical compounds, and a number of endogenous activators, e.g., reactive oxygen species, nitric oxide, and prostaglandins. The activation of TRPA1 results in the release of neuropeptides (e.g., substance P) from sensory neurons, resulting in neurogenic inflammation [29]. Additionally, TRPA1 mediates interleukin expression [30]. Another important ion channel family is P2 × 1-P2X7, activated by adenosine triphosphate (ATP). This group reacts to indirect injury mediated by ATP; it is involved in the sensation of visceral ad somatic pain associated with inflammation and nerve injury [25,27]. The transducer involved in the inflammatory nociception is acid-sensing ion channels (ASICs). ASIC activates nociceptors in the lowered pH environment in inflamed or injured tissue [25,27]. Ion channels are also responsible for signal transduction, as well as electrical transmission along axons, for example, voltage-gated Na channels (VGSCs, Na_v_), which play an important role in chronic neuropathies and sensitization of afferent neurons after nerve injury. VGSCs are also a target of anesthetic drugs, e.g., lignocaine [25,31]. VGSCs can be divided into two groups depending on their sensitivity to tetrodotoxin (TTX). TTX-resistant VGSCs are expressed in afferent fibers of sensory neurons: Na_v1.8_ in A fibers, and Na_v1.9_ in C fibers. Voltage-gated Ca^2+^ channels (VGCCs) are localized in the presynaptic membrane of dorsal root ganglion neurons and are responsible for the release of neurotransmitters [25,27]. VGCCs consist of three groups: the Cav1 family, the CaV2 family (including Ca_V_2.1—P/Q channels, Ca_V_2.2—N-type channels, Ca_V_2.3—R-type channels), and the Cav3 family (T-type channels). T-type Ca^2+^ channels are distributed in peripheral sensory neurons of dorsal root ganglion. The activation of these channels may be responsible for neuropathic pain processing [27].

GPCRs are cell-surface receptors that have an important role in the modulation of neuronal excitability. These groups of transducers include bradykinin receptors (B1, B2), prostaglandin E2 receptors (EP), and proteinase-activated receptors [25]. GPCRs in nociceptors may also have an inhibitory action on signal transmission, for example, opioid receptors [25]. The function of GPCRs on neuronal activity is conducted through various intracellular second-messenger pathways, including protein kinase A (PKA), protein kinase C (PKC), mitogen-activated protein kinase (MAPK), and intracellular calcium [32]. Activation of signaling pathways leads to a change in the activation of ion channels, for example TRPV1 and Na_V1.8_, which play an important role in signal transduction [25]. Cytokine receptors on afferent neurons also activate the intracellular signaling pathways, resulting in posttranslational changes in membrane transducers as well as transcriptional changes through transcription factors [33].

After nociceptor activation, the pain impulses are transmitted through two types of nerve fibers. Aδ myelinated fibers conduct fast and sharp sensations, while C demyelinated fibers transmit slower sensations [34]. These fibers end in the dorsal horn of the spinal cord, where the transmission continues through the second-order neurons to third-order neurons in the thalamus, where the pain is projected to specific cortical regions [22]. The modulation of pain is an endogenous mechanism that diminishes or enhances the transmission of pain [22]. Pain modulation may interact on various levels of nociception, e.g., on peripheral sensory neurons, the dorsal horn of the spinal cord and endogenous systems. Modulation of pain signals is mediated by segmental inhibition (gate theory), the endogenous opioid system, and descending inhibitory nerve system [22]. Segmental inhibition mechanisms include the blockade of the synapses between afferent nociceptive neurons and second-order neurons in the dorsal horn. This phenomenon may be present when myelinated nerve fibers Aβ sensing non-noxious stimuli (e.g., touch) stimulate the inhibitory nerve in the spinal cord, which blocks the transmission of the pain signal [35]. The endogenous opioid system comprises neurons that produce opioids: β-endorphin, enkephalins, and dynorphins. The endogenous opioids act as neuromodulators at three types of G-protein-coupled opioid receptors μ, δ, and κ [36]. The expression of these receptors has been found in the CNS (periaqueductal grey) and spinal cord (rostral ventromedial medulla) [22]. The activation of the μ opioid receptor is known for its analgesic effect. Κ and δ opioid receptors are responsible for pain modulation in peripherally mediated nociception [36]. Sensitization is an essential property of nociceptors to increase their excitability and decrease pain threshold, resulting in response to normally subthreshold stimuli [37]. Sensitization may regulate neuronal activity on both peripheral and central levels.

### 1.4. Pain Sensitization and Modulation

Peripheral sensitization occurs at the level of the primary sensory neuron [25]. It is mainly correlated with tissue injury or inflammation. It is caused by the inflammatory substances produced by inflammatory cells (e.g., mastocytes and macrophages), other nonneuronal cells (e.g., keratinocytes and fibroblasts), and sensory nerve fibers. These substances may directly activate nociceptors (e.g., protons and ATP) or mediate the sensitization through more complex pathways [38]. The mechanisms leading to increased excitability of afferent neurons include gene transcription changes, translation, and posttranslational changes [39]. Inflammatory markers produced on the injury site (e.g., bradykinin, histamine, and prostaglandins) affect peripheral nociceptors through activation of G-coupled receptors and the receptor tyrosine kinase that initiate intracellular signaling pathways resulting in PKA and PKC activation [32,38]. Those pathways lead to phosphorylation of neuronal intramembrane channels Nav1.8 and TRPV1 and are responsible for prolonged depolarization and increased response to stimuli [38].

Additionally, inflammatory stimuli through signaling pathways may regulate the transcription and translation of functional protein in the dorsal root ganglia neuron cell body, e.g., upregulation of the TRPV1 channel in response to inflammatory mediators [33]. Central sensitization is characterized by increased responsiveness of nociceptive neurons in the CNS [40]. It manifests as tactile allodynia, defined as pain evoked by a stimulus that is typically not painful in the absence of tissue injury or painful disorder. The mechanisms of this phenomenon include the amplification of spinal cord signaling; therefore, a non-noxious stimulus may elicit a painful sensation [38]. Central sensitization plays a vital role in neuropathic pain [41]. In the presence of nerve injury (Aδ and C fibers), second-order neurons in the spinal cord receive increased input from the periphery, which lead to central sensitization. In that state, non-noxious stimuli from Aβ fibers through hypersensitive second-order neurons may evoke pain [41]. It is hypothesized that central sensitization is caused by changes in the activation of the N-Methyl-D-Aspartate receptor (NMDA) and AMPA glutamate receptors. The alterations in receptors’ responsiveness may be caused by both trafficking of intracytoplasmic receptors to the membrane and phosphorylation of receptor/ion channel and changes in their properties and are responsible for sensitization. Accordingly, many factors released by the primary nociceptor may evoke central sensitization. In the dorsal horn of the spinal cord, glutamate (Glu), substance P (SP), and brain-derived neurotrophic factor (BDNF) are released from the primary nociceptor. Glu binds to AMPA and NMDA receptors, SP binds to the G-protein-coupled neurokinin one receptor (NK1R), and BDNF to the tyrosine kinase receptor (trkB) on the postsynaptic membrane. The binding of these neuromodulators to postsynaptic receptors increases intracellular Ca2+ levels and activates protein kinases (e.g., PKA and PKC). Activated kinases phosphorylate NMDA and AMPA receptors, resulting in increased neural sensitivity [42].

There are several substances necessary in the transduction and translation of pain. One of the essential neuropeptides in the nociceptive process is SP. It is responsible for transmitting nociceptive signals through primary afferent fibers to second-order spinal neurons [43]. SP is synthesized in dorsal root ganglion neurons and is released during painful stimulation from peripheral and spinal terminals. In the spinal cord, it acts on nociceptive transmission through binding to the NK1R G-protein-coupled receptor. Another pathway includes the co-release of Glu from SP-positive nociceptors and the promotion of nociceptive sensitization [44].

Bradykinin is an inflammatory mediator that sensitizes peripheral nociceptors by reducing the pain threshold. Bradykinin is released from kininogen at the site of inflammation by proteases and acts on B_2_ and injury-induced B_1_ G-protein-coupled receptors on primary afferent fibers and contributes to peripheral sensitization [45].

**Figure 1:**
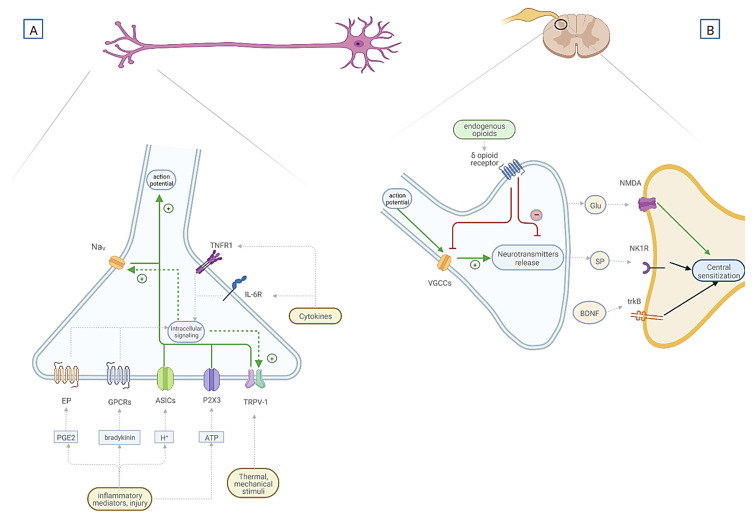
General mechanisms of nociception and sensitization. (**A**) Primary sensory neuron and surface receptors responsible for pain transduction, sensitive to various stimuli. Transient receptor potential subfamily V (TRPV1) is sensitive to thermal and mechanical stimuli. Ion channels—acid-sensing ion channels (ASICs) and P2X3—are activated by protons and ATP, respectively. G-protein-coupled receptors (GPCRs), including prostaglandin E2 receptors (EP), are sensitive to various inflammatory mediators, e.g., bradykinin and prostaglandin E2 (PGE2). The interaction between inflammation and nociception may occur through proinflammatory cytokines, e.g., interleukin-6 (IL-6), tumor necrosis factor α (TNFα), and their receptors (IL-6R, TNFR1), as well as other inflammatory mediators and their receptors—GPCRs or ion channels. The activation of TNF1R and IL-6R through various intracellular signaling pathways enhances translation and upregulation of TRPV1, leading to neuronal sensitization. Ion channels may evoke action potential by direct depolarizing neuronal cell membrane to initiate a nociceptive signal. GPCRs activation results in posttranslational changes in ion channels, e.g., TRPV-1 and voltage-gated Na channels (Na_V_), leading to prolonged depolarization and increased response to stimuli. Nav channels play a crucial role in signal transmission along neuronal fibers. (**B**) Dorsal horn of spinal cord, mechanisms of pain transmission, modulation, and central sensitization. The synapse is between the primary sensory neuron and second-order neuron in the spinal cord. On the presynaptic membrane of a primary sensory neuron are localized voltage gated Ca^2+^ channels (VGCCs) and opioid receptors. Activation of VGCCs is responsible for the release of neurotransmitters, e.g., glutamate (Glu) and substance P (SP). Endogenous opioids binding to opioid receptors on the presynaptic membrane negatively modulate neurotransmitters release by inhibiting VGCCs. The mechanism of central sensitization is caused by changes in the activation of the N-Methyl-D-Aspartate receptor (NMDA) and factors released by primary nociceptors, e.g., Glu, SP, and brain-derived neurotrophic factor (BDNF). Glutamate binds to NMDA receptors, SP binds to the G-protein-coupled neurokinin one receptor (NK1R), and BDNF to the tyrosine kinase receptor (trkB) on the postsynaptic membrane. The binding of these neuromodulators to postsynaptic receptors results in increased neural sensitivity. Legend: ASICs—acid-sensing ion channels; ATP—adenosine triphosphate; BDNF—brain-derived neurotrophic factor; EP—prostaglandin E2 receptor; Glu—glutamate; GPCRs—G protein-coupled receptors; H^+^—protons; HIF-1α—hypoxia-inducible factor 1α; IGF-1—insulin growth factor 1; IL-6—interleukin 6; Na_V_—voltage-gated Na channels; NH—nocturnal hypoxemia; NK1-R—neurokinin receptor; NMDA—N-Methyl-D-Aspartate receptor; OSA—obstructive sleep apnea; PGE2—prostaglandin E2; P2X3—purinergic receptors; SP—substance P; TNFα—tumor necrosis factor α; TNFR1—TNFα receptor; trkB—tyrosine kinase receptor for BDNF; TRPV1—transient receptor potential subfamily V; VGCCs—voltage-gated Ca channels. Created with BioRender.com. accessed on 13 July 2022.

Prostaglandin E2 (PGE2) is a lipid inflammatory mediator that plays a vital role in acute inflammatory pain. EP receptors activate TRPV1 channels, P2X3 receptors, and voltage-gated calcium or sodium channels in primary nociceptors, leading to hyperalgesia [46]. It is hypothesized that prolonged sensitization of dorsal root ganglion neurons may transition from acute to chronic pain [47]. (See Figure 1 for the graphical summary of the general mechanisms of pain transduction, transmission and modulation.)

### 1.5. OSA and Pain

Recent studies describing the link between OSA and pain provide evidence about the influence of polysomnographic (PSG) variables, sleep fragmentation, or continuous positive airway pressure (CPAP) therapy implementation on pain outcomes and analgesic therapy.

Few authors provide evidence that nocturnal oxygen desaturation among OSA patients is associated with higher analgesic sensitivity to opioids [4]. A similar correlation has been observed for higher serum levels of the hypoxia marker insulin growth factor binding protein-1 (IGFBP-1) [48], as well as for proinflammatory interleukins [4]. Another observed effect of nocturnal oxygen desaturation is increased odds of morning headache, headache disrupting sleep, and chest pain while in bed (pain outcome assessed with numerical pain scales) among OSA patients [7]. Authors of an experimental study have observed that mean nocturnal saturation (mean SaO_2_) is significantly correlated with decreased pressure pain tolerance (measured with a dolorimeter) [49]. A further step is determining the role of CPAP therapy, as the standard treatment of OSA [50], in pain alleviation. Experimental laboratory pain testing on a group of patients undergoing CPAP therapy resulted in increased finger withdrawal latency (FWL), a method used to assess the pain threshold to radiant thermal stimuli [8]. Additionally, after 2-night discontinuation of CPAP treatment, FWL decreased; this may lead to the conclusion that CPAP therapy successfully reduces pain sensitivity in OSA patients [51].

The impact of pain on OSA has been widely discussed. There are some papers describing the influence of pain and opioid therapy on sleep architecture, worse sleep quality and disordered breathing during sleep [52,53]. This correlation has been described in several studies; therefore, in our review we would like to focus on the effect of OSA and its pathophysiology on pain.

In this paper, we aimed to summarize the knowledge regarding the impact of OSA on pain perception and modulation, and further, to explain the relationship between those two on a clinical and molecular level with the division into inflammation, hypoxia, opioid receptors, and function of BDNF in OSA.

## 2. OSA, Inflammation, and Pain

One of the most well-known pain-inducing mechanisms is inflammation. There are plenty of described pathways connecting ongoing inflammation and the onset of pain sensation, one of them being the release of the inflammatory mediators and their effect on the signal transmitting properties of nociceptors (see Figure 1) [54].

The mechanism of inflammatory cytokines’ pronociceptive function varies depending on the nervous system’s anatomical level [33]. Peripheral nociceptors are the most vulnerable to circulating cytokines due to proximity to many immune cells (e.g., macrophages, neutrophils) that promote modulation of nociceptor function [33,55]. In mouse models with experimental inflammatory pain, rodent recombinant tumor necrosis factor α (TNFα) has been described to act on sensory neurons and induce hyperalgesic sensitization [56]. TNFα is an inflammatory cytokine involved in the evolution and persistence of inflammatory pain [57]. It acts on neurons and glial cells through two surface receptors, TNFR1 and TNFR2, to activate the NFκB signaling cascade, which may lead to the expression of a variety of proteins involved in neuronal sensitivity, e.g., cyclooxygenase 2 (COX2) and inducible nitric oxide synthase [58,59]. The TNFα—TNFR1 complex is also suggested to activate protein kinase C ε (PKCε), which evokes hyperalgesic priming (sensitization) in primary afferent nociceptors [56]. Another inflammatory cytokine to play an essential role in peripheral pain mechanisms is interleukin-6 (IL-6). IL-6 acts on nociceptors through the IL-6 receptor (IL-6R), which leads to dimerization of glycoprotein 130 and activation of the intracellular cascade signaling system through Janus kinase (JAK), signal transducers and activators of transcription (STATs) [60]. The alternative signaling pathway goes through a Ras/Raf-dependent MAPK cascade, influencing gene expression. IL-6 can activate neurons through membrane-bound (classic signaling) and soluble forms of IL-6R (trans-signaling) [61]. IL-6 trans-signaling can activate primary afferent sensory neurons through enhanced translation and upregulation of proteins involved in neurotransmission, e.g., TRPV1 and TRPV2 ion channels [62,63].

**Figure 2:**
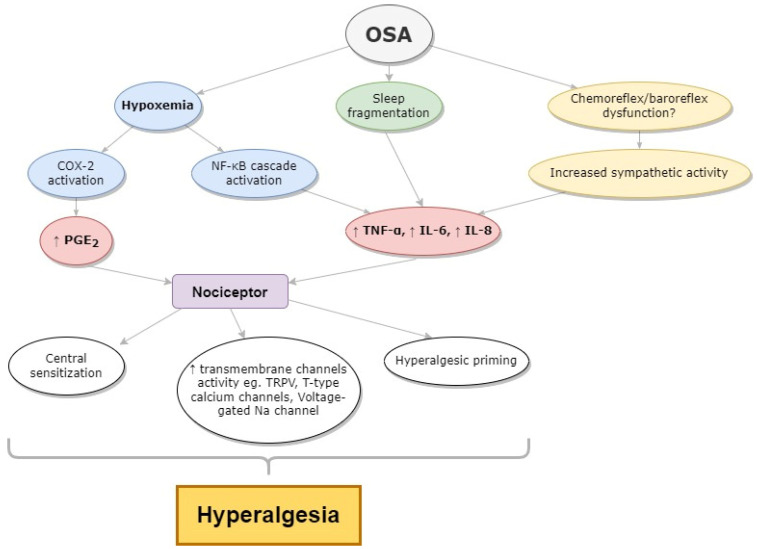
The influence of inflammatory markers in OSA on hyperalgesia. Legend: COX-2—cyclooxygenase—2: IL-6—interleukin—6; IL-8—interleukin-8; OSA—obstructive sleep apnea; PGE2—prostaglandin E2; TNFα—tumor necrosis factor α; TRPV—transient receptor potential subfamily V. The arrows represent cause and effect sequence.

The link between OSA and systemic inflammation has been a topic of interest and has been widely described in recent years [64,65,66]. It is commonly described that OSA is linked with increased expression of inflammatory mediators and elevation of many pro-inflammatory cytokines (PIC) [64]. (Figure 2) The most documented are IL-6 and TNFα [67,68]. Moreover, increased IL-6 and TNFα serum level is correlated with sleep fragmentation and hypoxia [69,70]. Another well-known cytokine involved in the proinflammatory reaction in OSA is IL-8. Its increased levels are observed in both adult and pediatric populations of OSA patients [71]. Elevation of PIC might have different pathogenetic causes. One might be the activation of the NFκB transcription factor in hypoxemic conditions [72]. NFκB plays a vital role in the stimulation of cytokine production, especially TNFα and IL8 [72]. Another proposed proinflammatory mechanism in OSA is sympathetic nervous system (SNS) activation [73]. Possible pathways leading to increased sympathetic activity include chemoreflex and baroreflex dysfunction. Increased SNS activation in OSA patients probably results from disturbed nocturnal breathing when repetitive obturations of upper airways induce hypoxemia and hypercapnia, which act through chemoreflex on sympathetic activity. A decreased baroreflex sensitivity may be provoked by repetitive blood pressure surges during sleep and increased SNS activation [74,75]. Some authors have proven that increased SNS activation leads to elevated interleukin concentrations in OSA patients [76] (Figure 2).

Sleep fragmentation and hypoxia are the main consequences of OSA. Sleep fragmentation often results in loss of total sleep time. It has been observed among healthy adults that sleep loss at the level of 4 h per night for ten days can induce the elevation of plasma levels of IL-6 and is correlated with exacerbated pain sensation and increased overall bodily discomfort [77]. That study also provides evidence that the association between physical discomfort and increased levels of IL-6 remains significant after controlling for the influence of fatigue [77]. That fact may suggest that IL-6–pain correlation in the case of prolonged sleep deprivation is not mediated through the changes in fatigue/tiredness, although presumably through the molecular interactions between IL-6 with nociceptive neurons.

Additionally, the elevation of genomic markers of inflammation, such as IL-6 messenger RNA and TNFα mRNA, has been observed in sleep loss conditions [78]. That proves the hypothesis that sleep deprivation conditions lead to upregulation of PIC gene expression and functional alteration of the monocyte inflammatory response. Furthermore, in the same study, the authors also provided bioinformatic analyses suggesting that the inflammatory response after partial sleep deprivation was mediated by the nuclear factor κB (NF κB) inflammatory signaling system and through classic hormone and growth factor response pathways [78].

Nocturnal hypoxia also plays a role in the upregulation of PIC levels, especially IL-6 [79]. One study showed that nocturnal hypoxia and body mass index (BMI) were the primary factors influencing IL-6 levels. Additionally, the authors provided evidence that nasal continuous positive airway pressure (nCPAP) therapy significantly lowered levels of IL-6 and spontaneous IL-6 production by monocytes [79]. It is also proven that intermittent hypoxia in OSA is responsible for selective activation of inflammatory pathways such as NFκB, which plays a central role in the inflammatory response and regulates the expression of cytokines, mainly TNFα and IL-6 [80].

PICs such as TNFα, IL-6, and IL-1β may modulate and increase pain sensation. The influence of PICs on the sensitization of peripheral nociceptors is a well-known process. The mechanism of this hyperalgesic effect of PICs may be through, for example, the enhancement of TRPV1 and TRPV2 channels activity, which may lower the neuronal response threshold (see Figure 1) [62,63,81].

There is some evidence that IL-6 and TNFα induce central sensitization and, therefore, hyperalgesia by an increase in excitatory synaptic mechanisms and decrease in inhibitory synaptic transmission through enhancing AMPA- or NMDA-induced currents by TNFα and IL-1β, and suppression of GABA- and glycine-induced currents by IL-1β and IL-6 in superficial dorsal horn neurons [82]. Central sensitization is involved in the induction and maintenance of chronic pain [83].

OSA-related intermittent hypoxia is also responsible for activating the COX-2 pathway and increasing PGE2 serum levels [84]. PGE2 binds to EP1 and EP4 receptors on primary sensory neurons, resulting in the intracellular activation of PKC and PKA, respectively [46]. PKC and PKA are responsible for activating neuronal transmembrane molecules such as TRPV1 channels, purinergic P2X3 receptors, Ca v3.2 T-type calcium channels, and voltage-gated sodium channels, resulting in inflammatory hyperalgesia [46].

Another interesting neuropeptide involved in the regulation of inflammation and nociception is calcitonin gene-related protein (CGRP). CGRP is a neuropeptide mainly produced and stored in the nervous system. CGPR exists in two isoforms—αCGRP and βCGRP. Both isoforms share similar biological functions, although they differ in their distribution in the nervous system. αCGRP has been found mainly in the central and peripheral nervous system, while βCGRP has been found in the enteric nervous system [85]. The synthesis of CGRP is upregulated in nerve damage models and through increased nerve growth factor (NGF) or BDNF concentration [86,87]. CGRP is stored in sensory neuron terminals and released after neuronal depolarization through calcium-dependent exocytosis. the TRP channel family has been widely described to have an impact on increased CGRP release from sensory neurons, especially the activation of TRPV1 and TRPA1, which play an important role in inflammatory pain transduction. CGPR is mainly localized in sensory unmyelinated C-fibers and myelinated Aβ fibers, especially on perivascular nerves [88]. The CGRP receptor consists of two subunits—calcitonin receptor-like receptor (CLR) and receptor activity modifying protein (RAMP). Activation of the CGRP receptor mediates various intracellular pathways resulting in, e.g., activation of potassium-sensitive ATP channels, an increase in intracellular calcium concentration and increased NO generation [85]. CGRP has various biological functions, including vasodilatation, modulation of the autonomic nervous system and transmission of the nociceptive signal. CGRP is also involved in neurogenic inflammation caused by the release of neuropeptides (CGRP, SP) from the nerve terminals, resulting in vasodilatation, edema, increased blood flow and recruitment of inflammatory cells in the affected site. The role of CGRP as an inflammatory mediator has been widely discussed. It has an impact on inflammatory cells’ activity, inhibits the proliferation and differentiation of lymphocytes and also has been suggested to promote inflammation through vasodilatation [85,89,90]. CGRP may also influence pain processing through its role in pain sensitization. It has been described that CGRP may have an impact on central sensitization while not on peripheral sensitization [91,92]. CGRP is suggested to have an important role in the development of inflammatory and neuropathic pain. In animal neuropathic models, the CGRP antagonist inhibited hyperalgesia, and a similar effect has been observed for CGRP knockout mice, which did not develop hyperalgesia after experimental inflammation [93,94,95]. Additionally, it has been described that in murine models, inflammation and increased levels of IL-1β and TNFα enhanced CGRP release [96]. These findings may lead to a conclusion that increased cytokine levels in OSA may possibly increase CGRP level and therefore induce inflammatory/neuropathic hypersensitization, although this hypothesis has not been researched yet. Further work in that field is needed to establish the correlation between OSA and CGRP-mediated pain sensitization.

## 3. OSA, Pain, and Hypoxia Markers

**Figure 3:**
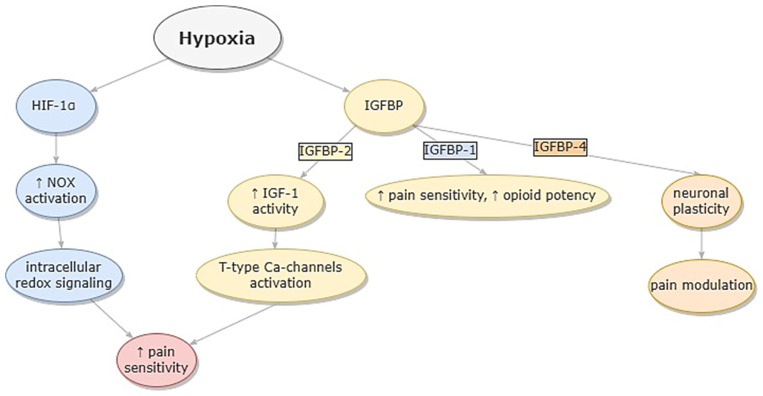
Influence of hypoxia markers in pain modulation in OSA. Legend: HIF-1α—hypoxia-inducible factor 1α; IGFBP—insulin growth factor binding protein; IGF-1—insulin growth factor—1; NOX—NADPH oxidases. The arrows represent cause and effect sequence.

The primary pathophysiological connection between OSA and pain refers to nocturnal hypoxemia and the role of hypoxia markers elevated in OSA in pain modulation (Figure 3).

Many physiological and pathophysiological changes in the course of OSA are related to hypoxemia, followed by activation of hypoxia-inducible factor (HIF) [97,98]. HIF is a transcription factor consisting of two subunits—stable β-subunit, constitutively expressed, and α-subunit, unstable under normoxia conditions and stable under hypoxia [17]. A stable dimeric complex of HIF interacts with gene promoters during hypoxemic conditions. It activates the expression of many genes responsible for, e.g., regulation of glucose metabolism [99], vessel growth, and the circadian clock [17,100,101]. In recent years, several papers have shown increased HIF-1α protein levels in OSA patients [102]. In this group of patients, HIF-1α is significantly elevated compared to the control group and presumably independent of the circadian rhythm [97,98,102]. Accordingly, another study provides evidence that apart from the stability of the increased level HIF-1α in OSA patients, HIF-1α protein concentration is chronically elevated and does not improve after one night of CPAP therapy [98].

Further research proves that in a group of OSA patients, after a follow-up of 2 months of nCPAP therapy, HIF-1α concentration decreased compared to the baseline [103]. Additionally, HIF-1α expression is positively correlated with the apnea–hypopnea index (AHI)—an index determining the severity of OSA [103]. The results of recent studies lead to a conclusion that chronic nocturnal hypoxemia in OSA causes the stable elevation of HIF-1α.

Among many physiological implications of upregulated activation of HIF-1α in hypoxemia, some authors also mention pain modulation. The exact role of nocturnal hypoxia and HIF-1α in pain perception is still discussed. Moreover, HIF-1α alternates cellular redox balance, which can lead to chronic pain development [104]. Overexpression of HIF-1α may be responsible for increased expression of the NADPH oxidases NOX4 and NOX 2 by enhancing NOX4 promoter activity by binding to the hypoxia-responsive element in the NOX4 promoter [105,106]. NOXs are also involved in nociceptive processing in neuropathic pain [107]. The pronociceptive role of NOX4 has been described as a result of overproduction of reactive oxygen species (ROS), in particular H_2_O_2_ and demyelination [107]. It is suggested that ROS may be involved in intracellular redox signaling in which H_2_O_2_ plays the role of the second messenger, reacting with protein cysteine residues and, through the process of thiol oxidation, resulting in the posttranslational formation of glutathionylated signaling proteins essential to redox signaling and sensitization of peripheral nociceptors [107,108,109]. This might be the possible pathway of the NOX4 selective contribution to neuropathic pain.

Additionally, NOX4-derived H_2_O_2_ appears responsible for peripheral nerve demyelination and neuropathic pain hypersensitivity, especially after nerve injury [107]. This fact may lead to the conclusion that HIF-1α could be an essential mediator of pain in hypoxemic conditions (Figure 3). The results of another study suggest that HIF-1α may have a dual impact on pain perception—it may alleviate acute pain. However, it may promote neuropathic pain through the activation of injured neurons [110], which is consistent with previous research in that field and brings new evidence to prove the hypothesis of HIF-1α involvement in neuropathic pain perception.

Another recently studied relation between HIF-1α and pain is TRPA1 expression regulation. It has been described that HIF-1α acts as a transcription factor on hypoxia-response elements such as the motif in *TRPA1* gene. The activation of the *TRPA1* promoter leads to increased TRPA1 expression [111]. The described pathway may be responsible for pain sensitization in OSA patients.

**Figure 4:**
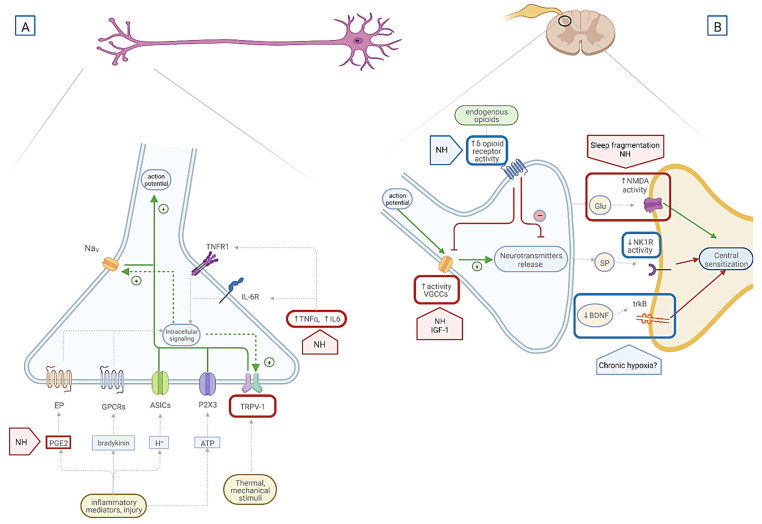
Interaction of OSA pathology on pain transmission and sensitization. (**A**) Pronociceptive function of pathophysiological changes in OSA are indicated in red brackets. Antinociceptive and modulatory functions of OSA are indicated in blue brackets. The green arrows indicate the activation function of receptors on signal transmission, the red arrows indicate suppression function on signal transmission. The grey arrows represent the influence of listed substance/receptor on receptor/intracellular signaling. The pro-inflammatory state caused by nocturnal hypoxemia manifests in increased serum levels of inflammatory mediators, e.g., prostaglandin 2 (PGE2) and cytokines—interleukin 6 (IL-6), tumor necrosis factor α (TNFα). Increased levels of these mediators act on primary sensory neurons through its receptors—TNFα receptor (TNFR1), interleukin-6 receptor (IL-6R), PGE2 receptor (EP), other G-protein-coupled receptors (GPCRs), and ion channel receptors (ASICs and P2 × 3 purinergic receptors). The activation of TNF1R and IL-6R through various intracellular signaling pathways enhances translation and upregulation of transient receptor potential subfamily V (TRPV1), leading to neuronal sensitization. Ion channels may evoke action potential by directly depolarizing neuronal cell membrane to initiate a nociceptive signal. GPCRs activation results in posttranslational changes in ion channels, e.g., TRPV-1 and voltage-gated Na channels (Na_V_), leading to prolonged depolarization and increased response to stimuli. (**B**) At the spinal cord level, nocturnal hypoxemia (NH) through elevated insulin growth factor 1(IGF-1) may be responsible for increased activation of presynaptic voltage-gated Ca channels (VGCCs) and therefore increased release of neurotransmitters such as glutamate (Glu) and substance P (SP). Nocturnal hypoxemia also increases N-Methyl-D-Aspartate receptor (NMDA) activity, which plays an important role in the mechanism of central sensitization. On the other hand, nocturnal hypoxemia by transcription factors such as hypoxia-inducible factor 1α (HIF-1α) increases the expression of opioid receptors and therefore inhibits pain signaling. Chronic hypoxia in OSA may decrease the activity of neurokinin receptor (NK1-R) and decrease serum level of brain-derived neurotrophic factor (BDNF)—factors responsible for central sensitization, and possibly reduce pain sensation. Trk b—tyrosine kinase receptor for BDNF. **Legend:** ASICs—acid-sensing ion channels; ATP—adenosine triphosphate; BDNF—brain-derived neurotrophic factor; EP—prostaglandin E2 receptor; Glu—glutamate; GPCRs—G protein-coupled receptors; H^+^—protons; HIF-1α; IGF-1—insulin growth factor 1; IL-6—interleukin 6; Na_V_—voltage-gated Na channels; NH—nocturnal hypoxemia; NK1-R—neurokinin receptor; NMDA—N-Methyl-D-Aspartate receptor; OSA—obstructive sleep apnea; PGE2—prostaglandin E2; P2 × 3—purinergic receptors; SP—substance P; TNFα—tumor necrosis factor α; TNFR1—TNFα receptor; trkB—tyrosine kinase receptor for BDNF; TRPV1—transient receptor potential subfamily V; VGCCs—voltage-gated Ca channels. Created with BioRender.com. https://biorender.com/ accessed on 13 July 2022.

The second well-known hypoxia marker is IGFBP-1 [112]. IGFBP-1 is responsible for the regulation of the bioavailability of insulin growth factor-1 (IGF-1), and it is proven to be correlated with hypoxemic states, especially in fetuses. The effect of IGFBP-1 on the nervous system has not been fully discovered yet. Some papers describe the effect of IGFBP-1 on brain development and impaired glial proliferation in response to injury in murine models [113,114]. While IGFBP-1 is not widely expressed in the nervous system, and its function results mainly from its endocrine process, other proteins from the IGFBP family are common in the central nervous system. It has been proven that IGFBP-2 is highly expressed by astrocytes in the cortex and has several functions, e.g., inhibition of oligodendrocyte precursors. Elevated membrane IGFBP-2 is also able to increase IGF-1 activity [115]. IGFBP-4 is involved in keeping up the cerebral plasticity and astrocyte microtubule function [113,116]. Higher serum IGFBP family protein concentration is correlated with OSA, especially IGFBP-4, which has been proven to be significantly elevated in a group of OSA patients [117]. IGFBP proteins are responsible for the regulation of IGF-1 activity, so presumably, they can affect the nociception through IGF-1 activation (see Figure 3). There is some evidence that elevated IGF-1 may enhance peripheral pain through the stimulation of T-type calcium channels in mouse models (see Figure 4) [118]. At the clinical level, it has been described that a change in serum-free IGF-1 in patients with fibromyalgia is correlated positively with changes in cerebrospinal fluid SP, neuropeptide Y level, and pain threshold [119]. This correlation could be possible in a group of OSA patients with chronic nocturnal hypoxemia and therefore elevated levels of IGFBP proteins responsible for overactivation of IGF-1, e.g., IGFBP-2. IGFBP is hypothesized to have a pronociceptive effect independently from IGF-1. One study presented results that IGFBP-1 is also correlated with pain regulation in OSA. However, it may decrease pain sensitivity and increase potency for opioid analgesia [4]; the same effect for opioid potency was observed for nocturnal hypoxemia measured as SaO2 nadir. The exact role of the IGF-IGFBP axis on pain perception in hypoxia conditions is not well understood yet and requires further research.

## 4. OSA and Opioid Receptors

OSA is not only responsible for hypersensitization of nociceptors, but it may also regulate the response to antinociceptive treatment. The correlation between OSA and opioid action is hypothesized to be bidirectional [120]. It has been described that opioid use may lead to sleep-disordered breathing, for example, through impaired upper airway function [121]. Studies provide evidence that μ-opioid receptor stimulation may suppress the hypoglossal motor nucleus and decrease the activity of the genioglossus muscle, leading to an increased risk of upper airway collapse [121,122]. However, the opinions on whether opioids can aggravate OSA remain ambiguous.

On the other hand, there is some evidence that hypoxemia in OSA patients is associated with reduced pain sensitivity and reduced opioid requirement for analgesia [123]. The effect of hypoxemic conditions in OSA on opioid function may apply to both the analgesic and respiratory depressant functions of opioids. One study provided evidence that nocturnal hypoxemia assessed by nadir SaO2 in OSA patients treated with fentanyl is correlated with a higher pain threshold than in the control group of patients without OSA [4]. These results are consistent with clinical studies conducted among the pediatric population, which showed that the group of patients with nadir SaO2 < 85% required a significantly lower analgesic morphine dose postoperatively [123]. The other experimental study shows that recurrent hypoxia in rats exposed to mu-opioid agonist fentanyl results in more profound depression of ventilation as a decrease in minute ventilation, respiratory frequency, and tidal volume [124].

The described phenomenon is possibly caused by increased expression of opioid receptors in CNS during hypoxia. In the study performed on a rat model, it was observed that the opioid receptors mu-opioid receptor (MOR) and delta-opioid receptor (DOR) were upregulated at both protein and mRNA levels after three weeks of hypoxia [125]. The possible pathway of opioid receptor mRNA upregulation in hypoxemic conditions includes activation of HIF-1α, which binds to gene promotor at a hypoxia-responsive element and results in transcription of MOR/DOR genes [125]. Increased expression of opioid receptors may be a possible reason for the observed hypoalgesic effect of nocturnal hypoxemia.

Another possible explanation of increased opioid sensitivity in OSA is an alteration of substance P/neurokinin 1 and the μ-opioid neuropeptide system in hypoxemic conditions. One experimental study on rats showed that exposure to single and prolonged sessions of intermittent hypoxia (IH) resulted in a reduction in ^125^I-substance P binding to the receptor, measured by quantitative film autoradiography [126]. This effect was not observed for the binding of the μ-opioid receptor agonist ^125^I DAMGO (Tyr-*d* -Ala-Gly-N-Me-Phe-Gly-ol), which was stable through the IH exposure [126]. The findings are related to the function of the substance P receptor NK1R and the μ-opioid receptor. Both receptors are G-protein-coupled receptors and are deactivated by internalization [127]. There is some evidence that the NK1R receptor is more prone to internalization than MOR, which is stable after activation by the ligand [128]. Some authors hypothesize that this imbalance in the activation of NK1R and MOR is even more significant under hypoxia [127]. This fact results in the increased binding of μ-opioid agonists to MOR and reduced binding of substance P to NK1R under hypoxia conditions [126] (Figure 4). The described situation results in the domination of μ-opioid and a decrease in substance P function. Substance P is documented to be an essential neurotransmitter involved in pain perception and additionally is proven to have an excitatory function in respiratory-related regions [129,130]. The tasks of μ-opioid agonists are opposite; the activation of MOR is implicated in antinociception and suppression of respiratory regions [131,132]. These results may lead to a conclusion that imbalance in substance P/neurokinin 1 and the μ-opioid neuropeptide system may be responsible for increased opioid sensitivity as well as for respiratory depressant function in OSA patients.

The described correlation between OSA, nocturnal hypoxemia and opioid sensitivity may be vital information for clinicians. Due to increased sensitivity to opioids in terms of analgesic effect and potentially increased risk of opioid-induced ventilatory impairment in OSA patients, it is important to properly adjust the dose of opioids in this group of patients. This clinical effect is especially important in the group of OSA patients undergoing postoperative analgesia, with a high risk of opioid-induced ventilatory impairment. It has been described that in this group of patients, several preventive procedures must be taken into consideration, e.g., minimizing perioperative opioid analgesia, and close monitoring of postoperative patients [133].

## 5. BDNF Role in OSA Neuronal Dysfunction

BDNF is a widely distributed neurotrophin in the central nervous system. BDNF is involved in many neurophysiological processes [134]. A variety of BDNF functions may be a result of its synthesis, which involves several active isoforms that could interact with different signaling pathways. BDNF plays a vital role in neuronal survival and developmental processes in the brain; it is responsible for the regulation of neuro, glio-, and synaptogenesis. Other functions include the control of synaptic interactions significant in memory and cognition mechanisms [134,135].

BDNF also regulates the nociceptive transmission through binding to the trkB receptor in the postsynaptic membrane in the spinal dorsal horn and may cause central sensitization [42] (Figure 4). The correlation between BDNF and OSA has not yet been fully understood, although some papers provide evidence that BDNF serum levels may be altered in OSA. The results of experimental studies show that exposure to acute hypoxia causes the elevation of serum BDNF concentration in animal models and in healthy adults [136,137]. This observation may lead to the conclusion that BDNF could be responsible for hypoxic preconditioning, a phenomenon occurring in hypoxic conditions to protect against ischemic brain damage [138]. On the other hand, a prolonged insult to the nervous system, such as chronic hypoxemia, may lead to decreased BDNF levels [139]. The results of an experimental study with a mouse model of OSA prove that chronic intermittent hypoxia is responsible for decreased BDNF expression and impaired hippocampal neuronal activity [140]. This observation is in line with the results of another animal model study, in which rats were treated with intermittent hypoxia [141]. The fact that BDNF is decreased in chronic hypoxemic states in OSA may lead to the hypothesis that intermittent hypoxia and decreased BDNF concentration could cause neurocognitive deficits in OSA [142]. The exact mechanism causing a BDNF decrease in chronic intermittent hypoxia is still unknown. The proposed pathway of this correlation includes activation of gene transcription, primarily via activation of the cAMP-response element-binding transcription factor. The authors of one study observed that the total production of cAMP-response element-binding protein (CREB)—the transcription factor responsible for BDNF gene transcription in hypoxic conditions—remains unchanged, while the phosphorylated form of CREB was significantly decreased after hypoxic treatment [139]. This observation may lead to the conclusion that BDNF may be affected by chronic IH at the gene level, which results in a decrease in BDNF expression. This result may form a hypothesis that as BDNF is a critical factor in the mechanism of central sensitization, its decreased expression under hypoxic conditions in OSA may negatively modulate pain transmission at the spinal level (Figure 4).

## 6. Conclusions

The results of recent studies on this topic prove the hypothesis that the complex pathophysiology of OSA plays an important role in pain regulation. However, there is still a discussion on the exact effect of each phenotype on pain sensation. Sleep fragmentation and sleep loss seem to enhance pain sensitivity through, e.g., inflammatory mediators [8,143,144]. For another component of OSA, nocturnal hypoxia, due to its complicated pathophysiological changes under hypoxemic conditions, it is difficult to assess the exact impact on painful sensation. There is some evidence that nocturnal hypoxemia may modulate opioid regulation of pain [4,145]. On the other hand, there are studies describing the hyperalgesic effect of hypoxemia [7]. The overall effect of OSA on pain is the sum of the influence of sleep loss, hypoxemia, inflammatory mediators, and hypoxia markers, e.g., HIF-1α, on the nociceptive neurons and on nociceptive transmission pathways. This may lead to the conclusion that the correlation of OSA and pain is complicated in terms of pathophysiology; therefore, more studies are needed to establish the mechanisms responsible for this interaction [146]. Considering the correlation between the proinflammatory cytokines, OSA, and pain, there is an important need for further research to establish possible therapeutic approaches that could be targeted to inflammatory signaling pathways, hypoxemic changes, and negative neuronal changes triggered by chronic hypoxia. Further research in that field is needed to answer how to break the pathophysiological pathways leading to the aggravation of chronic pain among OSA patients.

## Abbreviations

CGRP—Calcitonin gene related peptide	ASICs—acid-sensing ion channels
CNS—central nervous system	AHI—apnea-hypopnea index
CPAP—continuous positive airway pressure	ATP—adenosine triphosphate
COX-2—cyclooxygenase—2	B1—bradykinin receptor
CREB—cAMP response element-binding protein	BDNF—brain-derived neurotrophic factor
DOR—delta-opioid receptor	BMI—body mass index
MOR—mu-opioid receptor	EP—prostaglandin E2 receptor
OSA—obstructive sleep apnea	FWL—finger withdrawal latency
P2X—purinergic receptors	Glu—glutamate
PGE2—prostaglandin E2	Glu—glutamate
PIC—proinflammatory cytokines	GPCRs—G protein-coupled receptors
PKA—protein kinase A	HIF-1α—hypoxia inducible factor 1α
PKC—protein kinase C	IGF-1—insulin growth factor -1
PSG—polysomnography	IGFBP—insulin growth factor binding protein
ROS—reactive oxygen species	IL-6—interleukin 6
SaO2—oxygen saturation	IL-8—interleukin 8
SNS—sympathetic nervous system	IH—intermittent hypoxia
SP—substance P	JAK—Janus kinase
STATs—signal transducers and activators of transcription	MAPK—mitogen-activated protein kinase
TNFα—tumor necrosis factor α	Nav—voltage-gated Na channels
TNFR1—tumor necrosis factor α receptor	nCPAP—nasal continuous positive airway pressure therapy
TRPA1—transient receptor potential ankyrin 1	NH—nocturnal hypoxemia
TRPV—transient receptor potential subfamily V	NK-1R—neurokinin receptor
TTX—tetrodotoxin	NMDA—N-Methyl-D-Aspartate receptor
trkB—tyrosine kinase receptor	NOX—NADPH oxidases
VGSCs—voltage-gated Na channels	NF κB—nuclear factor κB
VGCCs—voltage-gated Ca2+ channels	Mean SaO2—mean oxygen saturation
